# Inverse association between estrogen receptor-α DNA methylation and breast composition in adolescent Chilean girls

**DOI:** 10.1186/s13148-018-0553-5

**Published:** 2018-10-04

**Authors:** Alexandra M Binder, Leah T Stiemsma, Kristen Keller, Sanne D van Otterdijk, Verónica Mericq, Ana Pereira, José L Santos, John Shepherd, Karin B Michels

**Affiliations:** 1 Department of Epidemiology, Fielding School of Public Health, University of California, Los Angeles, 90095 USA; 2 Department of Biostatistics, Fielding School of Public Health, University of California, Los Angeles, 90095 USA; 3 Institute for Prevention and Cancer Epidemiology, Faculty of Medicine and Medical Center, University of Freiburg, Freiburg im Breisgau, Germany; 4 Institute of Nutrition and Food Technology, University of Chile, Santiago, Chile; 5 Department of Nutrition, Diabetes and Metabolism, School of Medicine, Pontificia Universidad Católica de Chile, Santiago, Chile; 6 Population Sciences in the Pacific Program, University of Hawaii Cancer Center, Honolulu, HI 96813 USA

**Keywords:** Estrogen receptor-α, DNA methylation, Epigenetics, Breast density, Fibroglandular volume

## Abstract

**Background:**

Estrogen receptor-α (ER-α) is a transcriptional regulator, which mediates estrogen-dependent breast development, as well as breast tumorigenesis. The influence of epigenetic regulation of ER-α on adolescent breast composition has not been previously studied and could serve as a marker of pubertal health and susceptibility to breast cancer. We investigated the association between ER-α DNA methylation in leukocytes and breast composition in adolescent Chilean girls enrolled in the Growth and Obesity Cohort Study (GOCS) in Santiago, Chile. Breast composition (total breast volume (BV; cm^3^), fibroglandular volume (FGV; cm^3^), and percent fibroglandular volume (%FGV)) was measured at breast Tanner stage 4 (B4). ER-α promoter DNA methylation was assessed by pyrosequencing in blood samples collected at breast Tanner stages 2 (B2; *n* = 256) and B4 (*n* = 338).

**Results:**

After adjusting for fat percentage at breast density measurement, ER-α methylation at B2, and cellular heterogeneity, we observed an inverse association between B4 average ER-α DNA methylation and BV and FGV. Geometric mean BV was 15% lower (95% CI: − 28%, − 1%) among girls in the highest quartile of B4 ER-α methylation (6.96–23.60%) relative to the lowest (0.78–3.37%). Similarly, FGV was 19% lower (95% CI: − 33%, − 2%) among girls in the highest quartile of B4 ER-α methylation relative to the lowest. The association between ER-α methylation and breast composition was not significantly modified by body fat percentage and was not influenced by pubertal timing.

**Conclusions:**

These findings suggest that the methylation profile of ER-α may modulate adolescent response to estrogen and breast composition, which may influence breast cancer risk in adulthood.

**Electronic supplementary material:**

The online version of this article (10.1186/s13148-018-0553-5) contains supplementary material, which is available to authorized users.

## Background

Estrogen receptor-α (ER-α) is a ligand-activated transcriptional regulator, which mediates the action of estrogen and contributes to normal breast development and breast tumorigenesis [1]. Collectively, estrogen and its receptors (ER-α and β) regulate breast epithelial cell proliferation, differentiation, and apoptosis [1]. A majority of literature concerning ER-α is focused on its role in breast cancer; notably, two thirds of all breast cancer cases are associated with overexpression of ER-α [2, 3]. These ER+ tumors respond well to selective estrogen receptor modulators (e.g., tamoxifen), which competitively bind to estrogen receptors to prevent estrogen-dependent cancer growth [4]. DNA methylation of the ER-α promoter blocks the expression of ER-α [5–8]. Consequently, the DNA methylation profile of ER-α is currently being explored as a predictor of breast cancer incidence and prognosis (i.e., lower ER-α DNA methylation may be indicative of increased breast cancer risk). Due to its vital role in mediating the mammary tissue response to estrogen, ER-α methylation could also be considered as a potential marker of pubertal health, particularly in relation to mammary gland development.

ER-α plays a crucial role in mediating the action of estrogen in normal breast tissue and in regulating mammary gland development [9, 10]. ER-α knockout mice display decreased mammary epithelial cell proliferation and limited ductal growth [11, 12]. ER-α is also a major contributor to normal reproductive development [13, 14]. Knockout ER-α mice and mice with mutated ER- α are infertile and display a decreased response to estrogen [13, 14], suggesting normal ER-α expression is necessary to regulate the response to estrogen and guide normal pubertal development.

There have been no studies to date exploring the epigenetic regulation of ER-α in relation to adolescent breast development in humans. Variants in the ER-α gene (ESR1) have been associated with the increased percent fibroglandular volume (%FGV) in pre- and post-menopausal women [15–17], supporting a role of ER-α in regulating breast tissue composition in humans. The objective of this study was to analyze ER-α promoter DNA methylation in leukocytes during the pubertal time period (at breast Tanner stages 2 (B2) and 4 (B4)), in relation to total breast volume (BV), fibroglandular (FGV), and %FGV measured at B4 in a prospective cohort of girls enrolled in the Growth and Obesity Cohort Study (GOCS) in Santiago, Chile. Given the potential influence of the peripheral conversion of estrogen on ER-α regulation, the majority of which occurs in adipose tissue, we also evaluate potential effect modification of this relation by adolescent adiposity (body fat percentage) [18]. Further, we analyze effect modification by exposure to endocrine-disrupting chemicals (EDCs; phthalates and phenols), which mimic or antagonize the effects of endogenous hormones [19–21]. The methylation profile of ER-α in adolescence may represent an additional marker of pubertal health and a potential marker of breast cancer risk in adulthood.

## Results

### Demographics of Chilean girls enrolled in the Growth and Obesity Cohort Study

This study includes 429 Chilean girls enrolled in the Growth and Obesity Cohort Study (GOCS) and assessed for breast development at B4 (*n* = 345) and ER-α promoter DNA methylation at B2 (*n* = 256) and B4 (*n* = 338). The median age at methylation assessment was 10.1 years at B2 and 11.1 years at B4. It should be noted that the B2 assessment may not accurately represent age at thelarche, of which the median age was 9.3 years. The median age at menarche was 11.9 years. Body fat percentage was measured at B4, concurrent with breast composition measurements (median fat percentage = 26 %). The remaining demographics for this cohort are summarized in Table 1.

**Table 1:** Demographic characteristics of 429 Chilean girls participating in GOCS and included in this analysis

Covariate	Breast Tanner stage	Demographics	
Age at visit, years	B2	*n*	256
		Median	10.1
		25th–75th percentiles	9.4–10.8
	B4	*n*	358
		Median	11.1
		25th–75th percentiles	10.6–11.8
Fibroglandular volume (% DV/BV)	FGV	*n*	345
		Median	76.8
		25th–75th percentiles	58.7–98.4
	BV	*n*	345
		Median	200.5
		25th–75th percentiles	144.7–278.7
	%FGV	*n*	345
		Median	37.9
		25th–75th percentiles	27.2–53.4
Age at menarche, years		*n*	379
		Median	11.9
		25th–75th percentiles	11.2–12.4
Fat percentage at Tanner 4		*n*	357
		Median	26.0
		25th–75th percentiles	22.5–29.8
Maternal education	Completed secondary	*n*	328
		%	76.5
	Completed post-secondary	*n*	101
		%	23.5
ER-α methylation	B2	*n*	256
		Median	6.0
		25th–75th percentiles	4.0–8.8
	B4	*n*	338
		Median	7.0
		25th–75th percentiles	4.3–9.4

### ER-α methylation is moderately correlated between B2 and B4

ER-α methylation at B2 and B4 was assessed across 10 CpG sites (located within the 5′ untranslated region (5′UTR) of the *ESR1* gene) in blood samples from adolescent Chilean girls. Due to the high correlation in percent methylation across the interrogated CpG loci (Spearman rho = 0.65–98; Additional file 1: Figure S1), we summarized the methylation level of the ER-α promoter by the mean across all sites. Average ER-α methylation was moderately correlated between B2 and B4 (Spearman rho = 0.244, *p* = 0.002); the intra-individual correlation in methylation across Tanner stage was not improved after correction for cellular heterogeneity (Spearman rho = 0.215; *p* = 0.009). We also compared the level of methylation between B2 and B4 (paired *t* test) and found no statistically significant differences between these time points.

### ER-α methylation at B4 is inversely associated with B4 breast composition

**Fig. 1:**
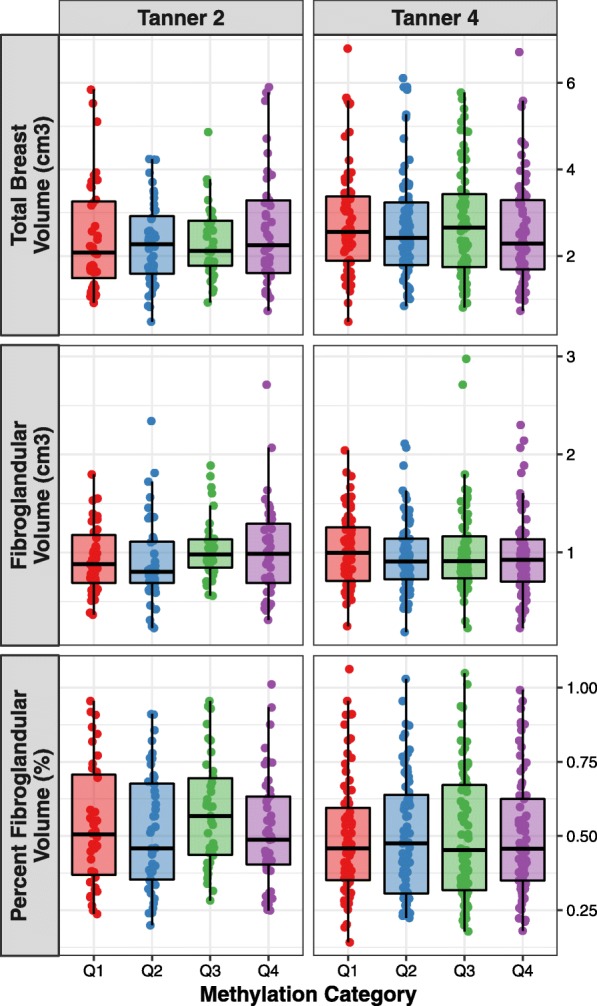
Breast composition by quartiles of average ER-α methylation. Quartiles for Tanner 2 methylation after correction for cellular heterogeneity: Q1 [0.98, 3.49], Q2 [3.49, 5.03], Q3 [5.03, 7.34], Q4 [7.34, 24.8]. Quartiles for Tanner 4 methylation after correction for cellular heterogeneity: Q1 [0.78, 3.37], Q2 [3.37, 5.28], Q3 [5.28, 6.96], Q4 [6.96, 23.60]

We first investigated the influence of ER-α methylation at B2 and B4 on adolescent (B4) breast composition, including total breast volume (BV; cm^3^), fibroglandular volume (FGV; cm^3^), and percent fibroglandular volume (%FGV). Average B2 ER-α methylation was not associated with any of these measures of breast composition in either unadjusted or adjusted models (Tables 2, 3, and 4). In contrast, we detected an inverse association between average B4 ER-α methylation and total BV, as well as FGV, after adjusting for B2 ER-α methylation, fat percentage at density measurement, and cellular heterogeneity (Fig. 1; Tables 2 and 3). Among girls in the same quartile of B2 ER-α methylation, those in the highest quartile (Q4) of B4 methylation (6.96–23.60%) had 15% lower (0.85; 95% confidence interval (CI): 0.72–0.99) geometric mean BV than girls in the lowest quartile (Q1) of B4 methylation (0.78–3.37%), adjusting for fat percentage and cellular heterogeneity. Similarly, geometric mean FGV was 19% lower (0.81; 95% CI: 0.67–0.98) among girls in the highest B4 methylation quartile relative to the lowest, adjusting for B2 ER-α methylation, fat percentage, and cellular heterogeneity. These associations were consistent after further adjustment for age at breast density measurement and maternal education (Tables 2 and 3). We observed a similar relation between B4 ER-α methylation and BV in models that were not adjusted for B2 ER-α methylation. However, these associations did not reach statistical significance (*p* > 0.05). Given the similar influence of B4 ER-α methylation on total BV and FGV, there was no association between ER-α methylation and %FGV. The impact of ER-α methylation on FGV and total BV at B4 did not related to age at menarche. Neither B2 nor B4 methylation was significantly associated with the timing of menarche, before or after adjustment for potential confounding variables (Table 5).

**Table 2:** ER-α methylation at B4 is inversely associated with B4 total BV in Chilean girls enrolled in GOCS

Stage label	Relative change in geometric mean BV (95% CI)		
	Model 1^a^	Model 2^b^	Model 3^c^
Tanner 2			
Quartiles^d^			
Q2	0.91 (0.79–1.05)	0.89 (0.76–1.03)	0.89 (0.77–1.04)
Q3	1.04 (0.90–1.21)	1.05 (0.90–1.22)	1.05 (0.90–1.22)
Q4	1.03 (0.89–1.19)	1.02 (0.88–1.19)	1.03 (0.89–1.20)
Linear model^e^			
	1.03 (0.96–1.09)	1.01 (0.95–1.08)	1.02 (0.95–1.09)
Tanner 4			
Quartiles^d^			
Q2	0.91 (0.82–1.00)	0.95 (0.86–1.06)	0.95 (0.86–1.06)
Q3	1.03 (0.93–1.14)	0.99 (0.89–1.10)	0.99 (0.89–1.10)
Q4	0.94 (0.85–1.04)	0.92 (0.83–1.02)	0.92 (0.83–1.02)
Linear model^e^			
	0.98 (0.94–1.03)	0.96 (0.91–1.00)	0.96 (0.91–1.00)
Tanner 2 (2 and 4)^f^			
Quartiles^d^			
Q2	0.89 (0.77–1.03)	0.90 (0.77–1.05)	0.90 (0.77–1.05)
Q3	1.07 (0.92–1.24)	1.08 (0.92–1.27)	1.08 (0.92–1.27)
Q4	1.00 (0.86–1.17)	1.02 (0.87–1.19)	1.01 (0.86–1.19)
Linear model^e^			
	1.02 (0.95–1.09)	1.00 (0.94–1.08)	1.00 (0.93–1.08)
Tanner 4 (2 and 4)^g^			
Quartiles^d^			
Q2	0.93 (0.81–1.07)	0.95 (0.82–1.10)	0.95 (0.82–1.10)
Q3	1.10 (0.94–1.28)	1.03 (0.88–1.21)	1.03 (0.88–1.21)
Q4	0.89 (0.77–1.04)	0.85 (0.72–0.99)*	*0.85 (0.72–0.99)* *
Linear model^e^			
	0.98 (0.91–1.04)	0.93 (0.86–1.00)*	0.93 (0.86–1.00)*

^a^Association with mean ER-α methylation adjusting for fat percentage at breast density measurement

^b^Association with cell composition corrected mean ER-α methylation adjusting for fat percentage at breast density measurement

^c^Model 2 additionally adjusted for age at breast density measurement and maternal education

^d^Quartiles for Tanner 2 methylation: Q1 [1.17, 4.05], Q2 (4.05, 6.04], Q3 (6.04, 8.85], Q4 (8.85, 29.30]; quartiles for Tanner 2 methylation after correction for cellular heterogeneity: Q1 [0.98, 3.49], Q2 (3.49, 5.03], Q3 (5.03, 7.34], Q4 (7.34, 24.8]. Quartiles for Tanner 4 methylation: Q1 [1.10, 4.27], Q2 (4.27, 7.05], Q3 (7.05, 9.37], Q4 (9.37, 32.00]; quartiles for Tanner 4 methylation after correction for cellular heterogeneity: Q1 [0.78, 3.37], Q2 (3.37, 5.28], Q3 (5.28, 6.96], Q4 (6.96, 23.60]

^e^Reporting relative change in geometric mean BV per doubling of percent methylation

^f^Modeling B2 and B4 ER-α methylation simultaneously, reporting association with B2 ER-α methylation

^g^Modeling B2 and B4 ER-α methylation simultaneously, reporting association with B4 ER-α methylation

*p<0.05, Wald test

**Table 3:** ER-α methylation at B4 is inversely associated with B4 FGV in Chilean girls enrolled in GOCS

Stage label	Relative change in geometric mean FGV (95% CI)		
	Model 1^a^	Model 2^b^	Model 3^c^
Tanner 2			
Quartiles^d^			
Q2	0.95 (0.80–1.12)	0.92 (0.77–1.10)	0.93 (0.77–1.11)
Q3	1.10 (0.92–1.31)	1.13 (0.94–1.37)	1.14 (0.95–1.37)
Q4	1.04 (0.88–1.25)	1.04 (0.87–1.24)	1.05 (0.88–1.27)
Linear model^e^			
	1.02 (0.95–1.10)	1.01 (0.93–1.09)	1.01 (0.94–1.09)
Tanner 4			
Quartiles^d^			
Q2	0.91 (0.80–1.03)	0.94 (0.83–1.07)	0.94 (0.83–1.08)
Q3	0.99 (0.87–1.12)	0.98 (0.86–1.11)	0.99 (0.87–1.13)
Q4	0.94 (0.83–1.07)	0.92 (0.81–1.04)	0.92 (0.81–1.05)
Linear model^e^			
	0.97 (0.92–1.03)	0.95 (0.90–1.01)	0.96 (0.90–1.01)
Tanner 2 (2 and 4)^f^			
Quartiles^d^			
Q2	0.93 (0.78–1.11)	0.92 (0.76–1.10)	0.92 (0.76–1.11)
Q3	1.14 (0.95–1.37)	1.20 (0.99–1.44)	1.20 (0.99–1.45)
Q4	1.02 (0.85–1.23)	1.05 (0.87–1.27)	1.05 (0.87–1.27)
Linear model^e^			
	1.02 (0.94–1.10)	1.01 (0.93–1.10)	1.02 (0.93–1.11)
Tanner 4 (2 and 4)^g^			
Quartiles^d^			
Q2	0.90 (0.76–1.06)	0.90 (0.76–1.07)	0.90 (0.76–1.07)
Q3	1.07 (0.89–1.28)	1.05 (0.87–1.26)	1.05 (0.87–1.27)
Q4	0.91 (0.76–1.09)	0.81 (0.67–0.98)*	0.81 (0.67–0.98)*
Linear model^e^			
	0.97 (0.90–1.05)	0.91 (0.83–0.99)*	0.91 (0.83–0.99)*

^a^Association with mean ER-α methylation adjusting for fat percentage at breast density measurement

^b^Association with cell composition corrected mean ER-α methylation adjusting for fat percentage at breast density measurement

^c^Model 2 additionally adjusted for age at breast density measurement and maternal education

^d^Quartiles for Tanner 2 methylation: Q1 [1.17, 4.05], Q2 (4.05, 6.04], Q3 (6.04, 8.85], Q4 (8.85, 29.30]; quartiles for Tanner 2 methylation after correction for cellular heterogeneity: Q1 [0.98, 3.49], Q2 (3.49, 5.03], Q3 (5.03, 7.34], Q4 (7.34, 24.8]. Quartiles for Tanner 4 methylation: Q1 [1.10, 4.27], Q2 (4.27, 7.05], Q3 (7.05, 9.37], Q4 (9.37, 32.00]; quartiles for Tanner 4 methylation after correction for cellular heterogeneity: Q1 [0.78, 3.37], Q2 (3.37, 5.28], Q3 (5.28, 6.96], Q4 (6.96, 23.60]

^e^Reporting relative change in geometric mean FGV per doubling of percent methylation

^f^Modeling B2 and B4 ER-α methylation simultaneously, reporting association with B2 ER-α methylation

^g^Modeling B2 and B4 ER-α methylation simultaneously, reporting association with B4 ER-α methylation

*p<0.05, Wald test

**Table 4:** ER-α methylation is not associated with B4 %FGV in Chilean girls enrolled in GOCS

Stage label	Relative change in geometric mean %FGV (95% CI)		
	Model 1^a^	Model 2^b^	Model 3^c^
Tanner 2			
Quartiles^d^			
Q2	1.04 (0.94–1.14)	1.03 (0.93–1.15)	1.04 (0.94–1.15)
Q3	1.05 (0.95–1.17)	1.08 (0.97–1.20)	1.08 (0.97–1.21)
Q4	1.01 (0.92–1.12)	1.01 (0.91–1.13)	1.02 (0.92–1.13)
Linear model^e^			
	1.00 (0.96–1.04)	1.00 (0.95–1.04)	1.00 (0.95–1.04)
Tanner 4			
Quartiles^d^			
Q2	1.01 (0.93–1.09)	0.99 (0.91–1.07)	0.99 (0.92–1.07)
Q3	0.96 (0.89–1.04)	0.99 (0.91–1.07)	1.00 (0.92–1.08)
Q4	1.01 (0.93–1.09)	1.00 (0.93–1.08)	1.01 (0.93–1.09)
Linear model^e^			
	0.99 (0.96–1.02)	1.00 (0.96–1.03)	1.00 (0.97–1.04)
Tanner 2 (2 and 4)^f^			
Quartiles^d^			
Q2	1.04 (0.93–1.15)	1.02 (0.91–1.14)	1.02 (0.91–1.14)
Q3	1.07 (0.95–1.19)	1.10 (0.99–1.23)	1.10 (0.98–1.23)
Q4	1.01 (0.91–1.13)	1.03 (0.92–1.15)	1.04 (0.93–1.16)
Linear model^e^			
	1.00 (0.95–1.05)	1.01 (0.96–1.06)	1.01 (0.96–1.06)
Tanner 4 (2 and 4)^g^			
Quartiles^d^			
Q2	0.97 (0.88–1.07)	0.95 (0.86–1.05)	0.96 (0.86–1.06)
Q3	0.98 (0.88–1.09)	1.02 (0.91–1.14)	1.02 (0.92–1.14)
Q4	1.02 (0.92–1.14)	0.96 (0.86–1.08)	0.96 (0.86–1.08)
Linear model^e^			
	1.00 (0.96–1.05)	0.99 (0.94–1.04)	0.99 (0.94–1.04)

^a^Association with mean ER-α methylation adjusting for fat percentage at breast density measurement

^b^Association with cell composition corrected mean ER-α methylation adjusting for fat percentage at breast density measurement

^c^Model 2 additionally adjusted for age at breast density measurement and maternal education

^d^Quartiles for Tanner 2 methylation: Q1 [1.17, 4.05], Q2 (4.05, 6.04], Q3 (6.04, 8.85], Q4 (8.85, 29.30]; quartiles for Tanner 2 methylation after correction for cellular heterogeneity: Q1 [0.98, 3.49], Q2 (3.49, 5.03], Q3 (5.03, 7.34], Q4 (7.34, 24.8]. Quartiles for Tanner 4 methylation: Q1 [1.10, 4.27], Q2 (4.27, 7.05], Q3 (7.05, 9.37], Q4 (9.37, 32.00]; quartiles for Tanner 4 methylation after correction for cellular heterogeneity: Q1 [0.78, 3.37], Q2 (3.37, 5.28], Q3 (5.28, 6.96], Q4 (6.96, 23.60]

^e^Reporting relative change in geometric mean %FGV per doubling of percent methylation

^f^Modeling B2 and B4 ER-α methylation simultaneously, reporting association with B2 ER-α methylation

^g^Modeling B2 and B4 ER-α methylation simultaneously, reporting association with B4 ER-α methylation

**Table 5:** ER-α methylation is not associated with age at menarche in Chilean girls enrolled in GOCS

Stage label	Relative time to menarche (95% CI)		
	Model 1^a^	Model 2^b^	Model 3^c^
Tanner 2			
Quartiles^d^			
Q2	0.98 (0.96–1.00)	0.99 (0.97–1.02)	1.00 (0.97–1.03)
Q3	0.99 (0.97–1.01)	0.98 (0.95–1.00)	0.98 (0.95–1.00)
Q4	0.98 (0.96–1.00)	0.98 (0.96–1.01)	0.99 (0.96–1.02)
Linear model^e^			
	0.99 (0.98–1.00)	0.99 (0.98–1.00)	0.99 (0.98–1.01)
Tanner 4			
Quartiles^d^			
Q2	1.00 (0.98–1.02)	1.01 (0.98–1.03)	1.01 (0.98–1.03)
Q3	0.99 (0.97–1.02)	0.99 (0.97–1.01)	0.99 (0.97–1.01)
Q4	1.00 (0.97–1.02)	1.01 (0.98–1.03)	1.01 (0.98–1.03)
Linear model^e^			
	1.00 (0.99–1.01)	1.00 (0.99–1.01)	1.00 (0.99–1.01)
Tanner 2 (2 and 4)^f^			
Quartiles^d^			
Q2	0.98 (0.95–1.01)	0.99 (0.96–1.03)	1.00 (0.97–1.03)
Q3	0.99 (0.96–1.02)	0.98 (0.94–1.01)	0.97 (0.94–1.01)
Q4	0.98 (0.95–1.01)	0.98 (0.95–1.01)	0.98 (0.95–1.01)
Linear model^e^			
	0.99 (0.98–1.01)	0.99 (0.98–1.00)	0.99 (0.98–1.00)
Tanner 4 (2 and 4)^g^			
Quartiles^d^			
Q2	1.00 (0.97–1.03)	1.01 (0.98–1.04)	1.01 (0.98–1.04)
Q3	1.01 (0.98–1.04)	1.00 (0.97–1.03)	1.00 (0.97–1.03)
Q4	0.99 (0.96–1.03)	1.01 (0.98–1.05)	1.01 (0.98–1.05)
Linear model^e^			
	1.00 (0.99–1.02)	1.01 (0.99–1.02)	1.01 (0.99–1.02)

^a^Relative time to menarche associated with mean ER-α methylation estimated via accelerated failure time model

^b^Association with cell composition corrected mean ER-α methylation

^c^Model 2 additionally adjusted for fat percentage at breast density measurement and maternal education

^d^Quartiles for Tanner 2 methylation: Q1 [1.17, 4.05], Q2 (4.05, 6.04], Q3 (6.04, 8.85], Q4 (8.85, 29.30]; Quartiles for Tanner 2 methylation after correction for cellular heterogeneity: Q1 [0.98, 3.49], Q2 (3.49, 5.03], Q3 (5.03, 7.34], Q4 (7.34, 24.8]. Quartiles for Tanner 4 methylation: Q1 [1.10, 4.27], Q2 (4.27, 7.05], Q3 (7.05, 9.37], Q4 (9.37, 32.00]; Quartiles for Tanner 4 methylation after correction for cellular heterogeneity: Q1 [0.78, 3.37], Q2 (3.37, 5.28], Q3 (5.28, 6.96], Q4 (6.96, 23.60]

^e^Reporting relative time to menarche per doubling of percent methylation

^f^Modeling B2 and B4 ER-α methylation simultaneously, reporting association with B2 ER-α methylation

^g^Modeling B2 and B4 ER-α methylation simultaneously, reporting association with B4 ER-α methylation

### Associations between ER-α methylation and breast composition are not modified by body fat percentage

We next evaluated whether the association between ER-α methylation and breast composition was modified by body fat percentage at B4. Percent body fat did not significantly modify the association between either B2 or B4 ER-α methylation and breast composition (results not shown). Likewise, percent body fat did not significantly interact with log-transformed ER-α methylation (%) to influence menarcheal age.

### Exposure to EDCs may modify the association between B2 ER-α methylation and B4 breast composition

**Fig. 2:**
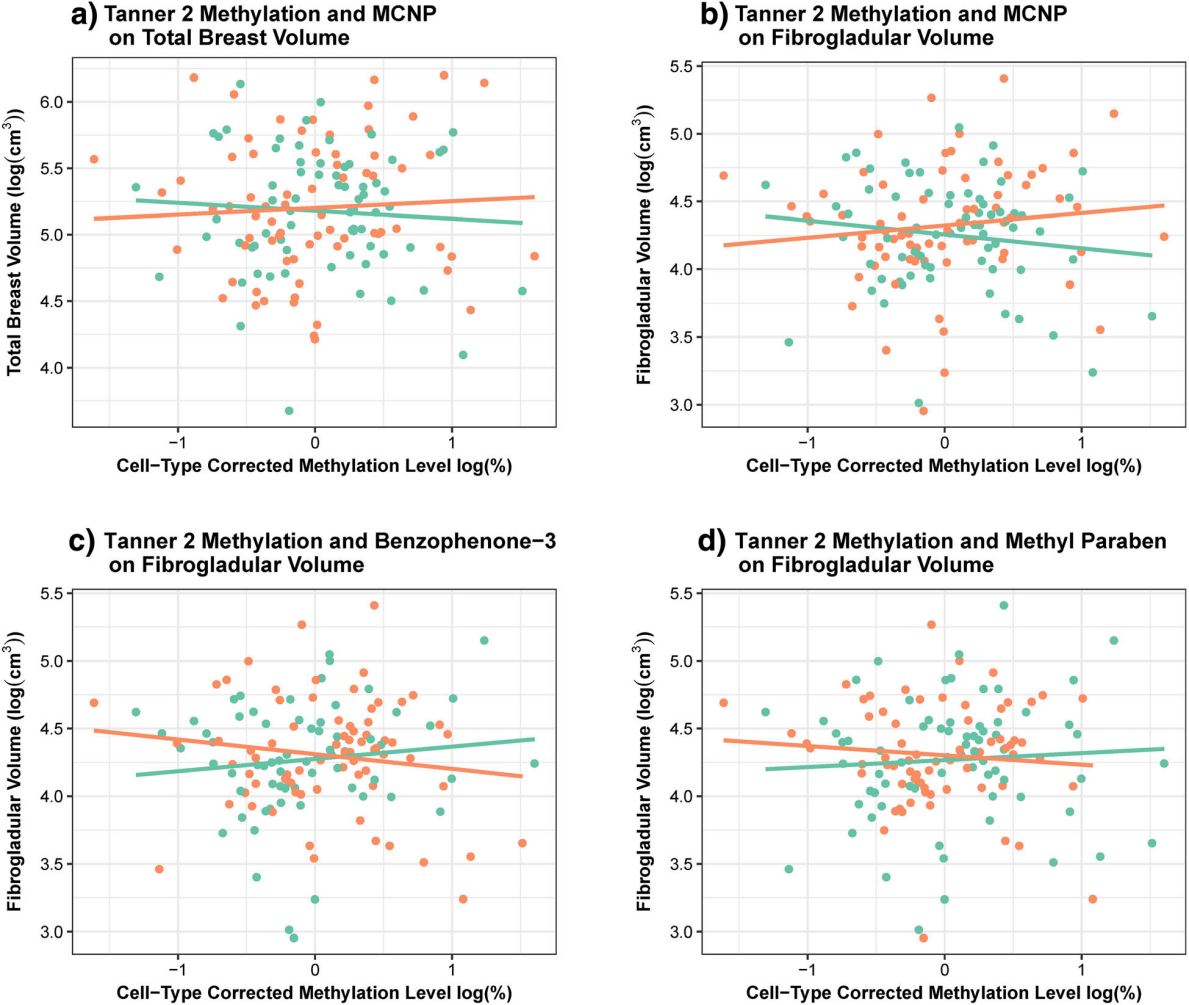
Associations between mean ER-α methylation and breast composition significantly (LRT, *p*<0.05) modified by adolescent EDC exposure. Plotting the association between log-transformed cell composition corrected Tanner 2 ER-α methylation and **a**) log-transformed BV, stratified by dichotomized MCNP levels; **b**) log-transformed FGV, stratified by dichotomized MCNP levels; **c**) log-transformed FGV, stratified by dichotomized benzophenone levels; **d**) log-transformed FGV, stratified by dichotomized methyl paraben levels. Average EDC concentrations were dichotomized by the median. Orange = high; green = low

Similarly, we postulated exposure to exogenous chemicals that mimic or antagonize the body’s endogenous hormones may modify the relation between ER-α methylation and breast composition. Urinary biomarkers of 26 phenols and phthalates were measured among 200 girls at B1 and B4; of these girls, DNA methylation results were available for 149 girls at B2 and 186 at B4. We previously observed no statistically significant difference in the influence of B1 and B4 phenol and phthalate concentrations on adolescent breast density in this population [publication accepted - in process]. Biomarker concentrations of monocarboxyisononyl phthalate (MCNP) significantly modified the influence of log-transformed B2 ER-α methylation on BV (likelihood ratio test (LRT), *p* = 0.036) and FGV (LRT, *p* = 0.014). The association between B2 ER-α methylation and FGV was also modified by average levels of both benzophenone-3 (LRT, *p* = 0.023) and methyl paraben urinary concentration (LRT, *p* = 0.041). In each of these cases, the impact of B2 ER-α methylation on either BV or FGV appeared to be in opposite directions for girls with low biomarker concentrations compared to those with high biomarker concentrations (Fig. 2; Table 6). However, none of these associations, stratified by dichotomized EDC category, reached statistical significance (Table 6; *p* > 0.05). The influence of B4 ER-α methylation on the breast measurements was not significantly modified by any of the measured EDCs. While we did detect a significant interaction between propyl paraben and log-transformed B2 ER-α methylation on the timing of menarche, the interaction was no longer statistically significant after the exclusion of one influential observation. We note that these associations should be interpreted with caution given the limited power to account for the role of type-I error inflation.

**Table 6:** Relative change in geometric mean B4 breast composition (95% CI) associated with a doubling of cell composition corrected mean Tanner 2 ER-α methylation that is significantly (LRT, *p*<0.05) modified by specific EDC biomarker concentrations

Outcome			EDC category^b^	
	EDC	LRT *p* value^a^	High	Low
Total breast volume (cm^3^)				
	MCNP	0.036	1.03 (0.93–1.14)	0.97 (0.87–1.09)
Fibroglandular volume (cm^3^)				
	MCNP	0.014	1.06 (0.95–1.19)	0.93 (0.82–1.07)
	Benzophenone-3	0.023	0.93 (0.82–1.05)	1.07 (0.94–1.21)
	Methyl paraben	0.041	0.95 (0.83–1.09)	1.04 (0.93–1.17)

^a^Likelihood ratio test (LRT) p-value comparing a model for log transformed breast composition that includes log-transformed cell composition corrected mean ER-α methylation, log-transformed EDC biomarker concentration, fat percentage and age at breast density measurement, and maternal education, to a model that additionally includes an interaction between log-transformed methylation level and log transformed EDC biomarker concentration. Restricting to models for which the statistical interaction term significantly (*p*<0.05) improved model fit

^b^Relative change in geometric mean breast composition associated with a doubling of percent methylation, stratifying by dichotomized (by the median) EDC metabolite concentrations, adjusting for fat percentage and age at breast density measurement, and maternal education

## Discussion

In this prospective cohort of Chilean girls, average B4 ER-α promoter DNA methylation was inversely associated with total BV and FGV measured at B4, adjusting for B2 ER-α methylation, cellular heterogeneity, and fat percentage at breast density measurement. Among individuals that were in the same quartile of B2 ER-α methylation, those in the lowest quartile of B4 ER-α methylation had greater BV and FGV than those in the highest quartile at B4. In other words, we observed a relative change in breast composition between groups that had the greatest divergence in ER-α methylation after B2. Due to similar associations with both total BV and FGV, epigenetic regulation of ER-α was not associated with %FGV in adolescence. Although ER-α has been reported to regulate reproductive development [13, 14], we did not observe it to be associated with menarcheal age. This could be attributed to the fact that endometrium growth is modulated by both ER-α and β [22, 23]. These findings were also not modified by adolescent body fat percentage, which suggests peripheral conversion of estrogen did not modulate the association between epigenetic regulation of its receptor and breast development at this stage. Lastly, we provide cursory evidence to support effect modification by exposure to specific EDCs on the relation between B2 ER-α methylation and B4 breast composition.

Promoter methylation of ER-α is highly correlated with its expression pattern [7, 8, 24, 25]. Accordingly, the observed inverse association between ER-α methylation and FGV and BV may reflect diminished sensitivity to estrogen-stimulated mammary epithelial cell proliferation and ductal growth [11, 12]. The association with ER-α methylation varied by developmental stage (B2 versus B4); only B4 ER-α methylation was associated with adolescent breast composition. Given B4 ER-α methylation and breast composition were measured concurrently, it is possible that decreased B4 ER-α methylation was a consequence of breast composition. However, mouse knockouts of *ERS1* suggest ER-α is a requisite for normal mammary gland and reproductive development [10, 26, 27]. Direct action of estrogen on its receptors initiates mammary epithelial cell proliferation by inducing expression of proliferative markers (e.g., Ki67). Subsequently, ER-α expression is reduced, while the proliferative markers remain expressed [10, 28], allowing for continued ductal growth. The inverse association at B4 is potentially due to continued downregulation of the *ESR1* gene. *ESR1* downregulation also correlates with the upregulation of inhibitory markers (e.g., TGF-β), which induce cell cycle arrest and may reduce epithelial proliferation in mammary tissue in the later stages of breast maturation [28–30]. Shepherd et al. report that the peak total FGV occurs at B4; hence, in this cohort, breast composition measurements were performed only at B4 [31]. Consequently, we cannot report on the association between B2 ER-α methylation and initiation of breast budding, beginning at B2. Perhaps we did not see an inverse association between B2 epigenetic regulation of ER-α and B4 breast composition due to the necessity for estrogen-stimulated mammary epithelial cell proliferation at the onset of ductal development. As breast maturation advances toward B4, however, there is a progressive reduction in ER-α expression associated with diminished mammary cell proliferation and enhanced ductal differentiation [28, 32]. Further, the B2 methylation assessment does not truly represent thelarche in this cohort and it is possible this may affect our analysis at B2.

We can only provide cursory evidence of the role of adolescent exposure to EDCs on breast development. However, exposure to high levels of EDCs appears to have differential effects on the relation between B2 ER-α methylation and BV and FGV. Among girls with high EDC exposure, we observed an inverse association between B2 methylation and FGV, suggesting that EDC exposure may decrease expression of ER-α and subsequently, FGV. The positive association between B2 ER-α methylation and BV among girls exposed to high levels of EDCs may be attributed to increased peripheral conversion of estrogen and subsequent increases in adiposity and BV.

Modulation of adolescent breast development by ER-α regulation could have future implications for breast cancer risk. Increased proportion of dense breast tissue (breast density, percent fibroglandular volume) in adults is one of the strongest and most consistent risk factors for breast cancer [33–35]. Peak breast density is postulated to be established during adolescence [36, 37], at which time the susceptibility of the developing mammary tissue to carcinogens is strongly enhanced. Correspondingly, Boyd and colleagues have speculated that women at high risk of breast cancer could be identified at an early age based on a breast density measurement [37]. The importance of pubertal development in breast cancer etiology is further highlighted by the inverse association between both age at breast bud development (thelarche) and age at menarche and breast cancer incidence [38–40]. In this study, epigenetic regulation of ER-α was not associated with age at menarche. This is perhaps due to our analysis of ER-α in blood, as pubertal timing is largely regulated by neuronal ER-α [41, 42]. The hypothalamic-pituitary-gonadal axis is also coordinated by many hormones (gonadotropic releasing hormone, testosterone, etc.) and endogenous and exogenous factors beyond estrogen [43], which may explain the lack of association between epigenetic regulation of ER-α and pubertal timing. Finally, it is possible this epigenetic signature is transient, instigating changes in pubertal breast development during a key developmental window of susceptibility for breast cancer. However, it is vital that ER-α methylation be assessed at additional time points throughout the life course to determine whether this methylation signature remains stable through post-pubertal mammary gland development.

In addition to addressing a major gap in our understanding of how ER-α regulation modifies adolescent breast development in humans, our study has a number of strengths. This investigation was conducted in a large, well-characterized longitudinal pediatric cohort. Assessment of ER-α methylation at two pubertal time points facilitated the identification of time-varying associations between epigenetic regulation of ER-α and breast composition. Measurement of body fat and urinary EDC biomarkers enabled examination of endogenous and exogenous modifiers of hormone regulation and potential impact on breast development. However, despite our relatively large sample size, after adjusting for multiple testing, we had limited power to identify significant interactions between EDC biomarker concentrations and ER-α methylation on breast composition. Another limitation of this study was the assessment of ER-α methylation in blood rather than breast tissue; however, in a pediatric cohort, biopsies of the target tissue (breast) are not feasible. Gene-specific methylation profiles are mildly correlated among whole blood and breast tumor samples; however, blood-derived DNA typically displays a lower methylation frequency for particular genes when compared to breast or tumor tissues [44, 45]. This limits the potential for ER-α blood DNA promoter methylation to serve as a surrogate marker of adolescent breast tissue methylation. However, as blood DNA methylation is currently being explored as a marker of breast cancer risk and breast tumor development [46], the blood epigenetic profile of ER-α may serve as a marker of adolescent breast composition. Additionally, due to our restriction to a Chilean cohort, we also note that the observed associations may not be generalizable to all populations as race/ethnicity may be associated with differential methylation profiles [47] and/or varied timing of breast maturation [48]. Finally, we do not yet know which of these girls may develop breast cancer in their lifetime, and thus, we cannot conclude that adolescent breast composition or the regulation of ER-α is predictive of breast cancer development in adulthood.

## Conclusion

Our study outlines the potential influence of ER-α epigenetic regulation on breast development in humans. Specifically, we identified an inverse association between increased ER-α DNA promoter methylation and total BV and FGV at B4 in Chilean girls. Future work in this research area should consider other endogenous and exogenous factors (e.g., dietary patterns) influencing adolescent ER-α methylation and implications for breast composition. To better characterize the role of ER-α in initiating breast development, researchers should also consider the analysis of the ER-α methylation profile and expression pattern at B1, prior to the onset of mammary gland development. Further, as breast cancer typically develops later in life, researchers should consider assessing ER-α methylation at later stages of development and during periods of significant hormonal shifts (B5, during pregnancy, and prior to menopause). Finally, it is important to note that follow-up proof-of-concept studies in humans and animal models are needed to verify our findings. For example, in vitro and/or in vivo experimental analyses should be applied to elucidate the feedback relation between estrogen levels and epigenetic regulation of ER-α during this critical window of breast development in adolescence. In addition, it will be important for future studies to assess the role of other biological pathways and genetic components, which may be modified by environmental factors and associated with pubertal development. In conclusion, this study greatly expands upon the current literature surrounding the role of ER-α in breast development by providing evidence in humans to support ER-α as a key modifier of pubertal breast composition and a potential risk marker for breast cancer in adulthood.

## Methods

### Study population

This study includes a subcohort of 429 Chilean girls enrolled in the Growth and Obesity Cohort Study (GOCS) with blood samples collected at B2 and/or at B4. Details of GOCS have been previously described [49]. Briefly, 1190 singleton children born at term (37–42 weeks) were enrolled in the study in 2006 when they were 2.6–4.0 years of age. All participants had a birth weight between 2500 and 4500 g. Children with any physical, medical, or endocrine diseases that might impact growth or puberty were excluded from the study. Children were physically assessed annually from 2006 to 2010 at the Institute of Nutrition and Food Technology Health Clinic in Santiago, Chile. From 2011 onward, the participants were physically assessed every 6 months. GOCS children were recruited from public nursery schools and are thus representative of low- to middle-income Chilean children from the southeast area of Santiago. The study protocol was approved by the Ethics Committee of the Institute of Nutrition and Food Technology, University of Chile. All parents and/or legal guardians gave signed informed consent prior to the data collection and children gave their assent.

### Dense breast tissue assessment

Assessment of breast density at B4 in GOCS has been previously described [36]. Briefly, breast development was assessed visually and by palpation by a single female trained dietitian (kappa with pediatric endocrinologist = 0.9) at clinical visits approximately every 6 months, beginning in 2009, using Tanner’s rating scale [50, 51]. At the first B4 visit, breast fibroglandular volume (FGV; cm^3^), total breast volume (BV; cm^3^), and percentage of fibroglandular volume (%FGV = FGV/BV × 100) were measured via dual-energy X-ray absorptiometry (DXA) in the left and right breasts. DXA has been previously validated and correlates strongly with mammography [31, 52, 53]. The dosage of radiation exhibited by this assessment is extremely low, lower than that received during a transcontinental flight, limiting any significant health risks associated with this X-ray method [54]. Each breast was scanned using GE iDXA system software (version 13.6, GE Healthcare, Madison, WI, USA). Breast composition was derived from a two-compartment model of adipose and fibroglandular tissues using the software developed by Dr. Shepherd and colleagues, Department of Radiology and Biomedical Imaging, University of California, San Francisco (version 5). A quality control phantom that contained reference breast density materials was scanned throughout the study to ensure stable calibration. Values from the left and right breasts are averaged for all analyses.

### Biospecimen collection

Fasting blood samples were collected at B2 and B4 for ER-α methylation analysis. DNA was extracted from the blood leukocytes using the QIAamp DNA Blood Mini Kit (Qiagen). Fasting spot urine samples were collected between 10 AM and 12 PM in polypropylene sterile cups and were immediately vortexed and aliquoted. They were collected at breast Tanner stages 1 (B1) and B4 for analysis of exposure to EDCs.

### ER-α methylation analysis

#### Polymerase chain reaction (PCR)

Genomic DNA was treated with bisulphite salt using the EZ DNA Methylation-Gold kit (Zymo Research, Cat. No. D5007) according to the manufacturer’s protocol. DNA was amplified in 20-μl PCR reactions, containing 10 μl of Hot StarTaq Master Mix Kit (Qiagen, Cat. No 203446), 150 ng of each primer, and ~ 20 ng modified DNA. PCR was performed with one cycle of 95 °C for 15 min, 40 cycles of 95 °C for 30 s, 57–63 °C for 30 s, and 72 °C for 30 s, followed by one cycle of 72 °C for 10 min. The reverse primer included a 5′-biotin label to allow subsequent analysis by pyrosequencing. Primer sequences can be found in (Additional file 1: Table S1). PCRs were performed in duplicate using 96-well plates. Each PCR plate contained the participants’ DNA samples, as well as three dH_2_O samples as non-template controls and three samples with known methylation status as positive controls.

#### Pyrosequencing

Pyrosequencing was performed on a PyroMark Q24 MD pyrosequencer (Qiagen, Cat. No 9001514) according to the manufacturer’s recommendations. The primers were designed using the primer design program “PSQ assay” (Biotage), using the ER-α gene sequence that was obtained from the GenBank entry on NCBI (Additional file 1: Table S1).

Assay validation was carried out on samples of known methylation status, using the EpiTect Control DNA and Control DNA Set (Qiagen, Cat. No. 59568). All pyrosequencing analyses were performed in duplicate. If the duplicates of the individual samples showed a difference < 5% of methylation, the average methylation of the two measurements was used for further analyses. When the difference was > 5%, a third measurement was performed. Due to the high correlation in percent methylation across the 10 interrogated CpG loci, ER-α methylation was summarized by average methylation across pyrosequenced loci 1–8. Loci 9 and 10 were not included in this average due to low resolution at the end of the sequencing reads in approximately 50% of the samples. Average ER-α methylation independent of blood composition was estimated by regressing log-transformed ER-α methylation on the proportion of monocytes, basophils, eosinophils, neutrophils, and lymphocytes, stratified by breast Tanner stage at ER-α methylation measurement. The exponentiated residuals were used in subsequent models that we note were corrected for cellular heterogeneity.

### Endocrine-disrupting chemicals

Urinary biomarker concentrations of 26 phenols and phthalates were measured among a subset of 200 GOCS girls at breast B1 and B4. EDC assays were performed at the Centers for Disease Control and Prevention (CDC) National Center for Environmental Health Laboratory using previously described analytical methods [55, 56]. The analysis of blinded specimens by the CDC laboratory was determined not to constitute engagement in human subjects’ research. Concentrations below the limit of detection (LOD) were given an imputed value equal to LOD/sqrt(2). EDC biomarker concentrations (ng/ml) were corrected for specific gravity. Dilution adjustment was performed using the formula *P*_c_ = *P*[(1.015 − 1)/(SG − 1)], where *P*_c_ is the specific gravity-corrected biomarker concentration, *P* is the observed biomarker concentration*,* SG is the specific gravity of the urine sample, and 1.015 is the median SG of the study population [55–58]. The analysis was restricted to the subset of 21 EDCs for which biomarker concentrations were above the LOD in at least 75% of the samples. For this study, EDC measurements were averaged across B1 and B4.

### Age at menarche

Prior to the onset of B4, girls were asked to report their first menstrual bleeding at each 6-month visit. After achieving B4, girls were contacted by study dietitians every 3 months to survey whether the girl had reached menarche. During this phone interview, a questionnaire was used to differentiate menarche from other potential causes of vaginal bleeding, such as vaginal infection, urinary infection, or trauma. Longitudinal follow-up of participants enabled the confirmation of menarche onset.

### Covariates

Additional covariates included in this analysis were age at methylation assessment (B2 and B4), body fat percentage, and maternal education. All additional covariates were measured at B4. Maternal education status was self-reported by mothers at an in-clinic study visit and categorized as secondary or post-secondary for this study. Compared with BMI, body fat percentage is a more precise measurement of adolescent fat distribution. Fat percentage was estimated at each visit using Tanita-BC-418 MA bioelectrical impedance measurements (Tanita-Corporation, Tokyo, Japan), according to the manufacturer’s guidelines and at a measurement frequency of 50 kHz (accuracy 0.1 kg) [59].

### Statistical analyses

All breast measurements were log-transformed prior to analysis. Linear models were used to estimate the association between ER-α methylation and breast composition, adjusting for fat percentage at breast density measurement. We considered models additionally adjusted for cellular heterogeneity and further adjusted for age at breast density measurement and maternal education. We independently modeled the association between B2 average ER-α methylation and breast composition (*N* = 177; *N* = 164 correcting for cellular heterogeneity), as well as the association between average B4 ER-α methylation and breast composition (*N* = 329; *N* = 303 correcting for cellular heterogeneity). To identify time-dependent associations between ER-α methylation and breast composition, we also simultaneously modeled the influence of both B2 ER-α methylation and B4 ER-α methylation on these breast measurements (*N* = 161; *N* = 142 correcting for cellular heterogeneity). ER-α methylation was modeled both as quartiles and continuously as log-transformed percent methylation. Estimated associations and 95% confidence intervals (CI) between ER-α methylation and breast measurements were exponentiated to provide the percent change in geometric mean breast density measurement. When modeling methylation continuously, we report the percent change in geometric mean breast density measurement given a doubling in percent methylation. Accelerated failure time models were used to assess the influence of ER-α methylation on time to menarche, assuming a Weibull distribution. For incident cases, survival time was the age at menarche, estimated based on the time between the self-reported date of first menses and date of birth. Survival time for right-censored individuals was the age at last clinic visit, based on the time between the date of last visit and date of birth. Similar to the breast measurement models, time to menarche was modeled as a function of B2 ER-α methylation and B4 ER-α methylation both separately and together, adjusting for cellular heterogeneity. To assess whether B4 fat percentage significantly modified the association between log-transformed B2 ER-α methylation and breast measurement, we compared the fit of a model with and without an interaction term between these two dependent variables (likelihood ratio test (LRT)), adjusting for cellular heterogeneity. We similarly evaluated whether there was a statistically significant interaction between log-transformed average EDC biomarker concentration and log-transformed B2 ER-α methylation on breast measurement, adjusting for cellular heterogeneity and fat percentage. Each biomarker was modeled separately. Analogously, we analyzed effect modification by both fat percentage and EDC exposure on the association between B4 ER-α methylation and breast measurement, as well as on the impact of B2 ER-α methylation and B4 ER-α methylation on time to menarche, respectively. Among models significantly modified by EDC biomarker concentration (LRT, *p* < 0.05), the association between log-transformed ER-α methylation and breast measurement is reported stratified by dichotomized EDC level (high vs low relative to the median). All analyses were performed using R version 3.4.1 and visualized using ggplot2.

## Additional file

Additional file 1: **Figure S1.** Correlation across CpG sites at B2 and B4 (Spearman rho = 0.65–98). Table S1: Primer sequences and positions. (DOCX 592 kb)

## Abbreviations

%FGV: Percent fibroglandular volume
B1–B4: Breast Tanner stages 1–4
BMI: Body mass index
BV: Breast volume
CDC: Center for Disease Control
DXA: Dual-energy X-ray absorptiometry
EDC: Endocrine-disrupting chemical
ER-α: Estrogen receptor-alpha
FGV: Fibroglandular volume
GOCS: Growth and Obesity Cohort Study
LOD: Limit of detection
LRT: Likelihood ratio test
UTR: Untranslated region

## References

[1] Anderson E (2002). The role of oestrogen and progesterone receptors in human mammary development and tumorigenesis. Breast Cancer Res.

[2] Hagrass HA, Pasha HF, Ali AM (2014). Estrogen receptor alpha (ERalpha) promoter methylation status in tumor and serum DNA in Egyptian breast cancer patients. Gene.

[3] Williams KE, Anderton DL, Lee MP, Pentecost BT, Arcaro KF (2014). High-density array analysis of DNA methylation in tamoxifen-resistant breast cancer cell lines. Epigenetics.

[4] Davies C, Godwin J, Gray R, Clarke M, Cutter D, Darby S, McGale P, Pan HC, Taylor C, Early Breast Cancer Trialists’ Collaborative G (2011). Relevance of breast cancer hormone receptors and other factors to the efficacy of adjuvant tamoxifen: patient-level meta-analysis of randomised trials. Lancet.

[5] Mao X, Qiao Z, Fan C, Guo A, Yu X, Jin F (2016). Expression pattern and methylation of estrogen receptor alpha in breast intraductal proliferative lesions. Oncol Rep.

[6] Ung M, Ma X, Johnson KC, Christensen BC, Cheng C (2014). Effect of estrogen receptor alpha binding on functional DNA methylation in breast cancer. Epigenetics..

[7] Lapidus RG, Ferguson AT, Ottaviano YL, Parl FF, Smith HS, Weitzman SA, Baylin SB, Issa JP, Davidson NE (1996). Methylation of estrogen and progesterone receptor gene 5′ CpG islands correlates with lack of estrogen and progesterone receptor gene expression in breast tumors. Clin Cancer Res.

[8] Ottaviano YL, Issa JP, Parl FF, Smith HS, Baylin SB, Davidson NE (1994). Methylation of the estrogen receptor gene CpG island marks loss of estrogen receptor expression in human breast cancer cells. Cancer Res.

[9] Saji S, Jensen EV, Nilsson S, Rylander T, Warner M, Gustafsson JA (2000). Estrogen receptors alpha and beta in the rodent mammary gland. Proc Natl Acad Sci U S A.

[10] Cheng G, Weihua Z, Warner M, Gustafsson JA (2004). Estrogen receptors ER alpha and ER beta in proliferation in the rodent mammary gland. Proc Natl Acad Sci U S A.

[11] Mueller SO, Clark JA, Myers PH, Korach KS (2002). Mammary gland development in adult mice requires epithelial and stromal estrogen receptor alpha. Endocrinology.

[12] Haslam SZ, Woodward TL (2003). Host microenvironment in breast cancer development: epithelial-cell-stromal-cell interactions and steroid hormone action in normal and cancerous mammary gland. Breast Cancer Res.

[13] Sinkevicius KW, Woloszyn K, Laine M, Jackson KS, Greene GL, Woodruff TK, Burdette JE (2009). Characterization of the ovarian and reproductive abnormalities in prepubertal and adult estrogen non-responsive estrogen receptor alpha knock-in (ENERKI) mice. Steroids.

[14] Gieske MC, Kim HJ, Legan SJ, Koo Y, Krust A, Chambon P, Ko C (2008). Pituitary gonadotroph estrogen receptor-alpha is necessary for fertility in females. Endocrinology.

[15] Gomes-Rochette Neuza Felix, Souza Letícia Soncini, Tommasi Bruno Otoni, Pedrosa Diego França, Eis Sérgio Ragi, Fin Irani do Carmo Francischetto, Vieira Fernando Luiz Herkenhoff, Graceli Jones Bernardes, Rangel Letícia Batista Azevedo, Silva Ian Victor (2017). Association of PvuII and XbaI polymorphisms on estrogen receptor alpha (ESR1) gene to changes into serum lipid profile of post-menopausal women: Effects of aging, body mass index and breast cancer incidence. PLOS ONE.

[16] Souza MA, Fonseca Ade M, Bagnoli VR, Barros N, Neves EM, Moraes SD, Hortense VH, Soares JM, Baracat EC (2014). The expression of the estrogen receptor in obese patients with high breast density (HBD). Gynecol Endocrinol.

[17] Tchatchou S, Jung A, Hemminki K, Sutter C, Wappenschmidt B, Bugert P, Weber BH, Niederacher D, Arnold N, Varon-Mateeva R (2009). A variant affecting a putative miRNA target site in estrogen receptor (ESR) 1 is associated with breast cancer risk in premenopausal women. Carcinogenesis.

[18] Siiteri PK (1987). Adipose tissue as a source of hormones. Am J Clin Nutr.

[19] Markey CM, Luque EH, Munoz De Toro M, Sonnenschein C, Soto AM (2001). In utero exposure to bisphenol A alters the development and tissue organization of the mouse mammary gland. Biol Reprod.

[20] Rudel R (1997). Predicting health effects of exposures to compounds with estrogenic activity: methodological issues. Environ Health Perspect.

[21] Kuiper GG, Lemmen JG, Carlsson B, Corton JC, Safe SH, van der Saag PT, van der Burg B, Gustafsson JA (1998). Interaction of estrogenic chemicals and phytoestrogens with estrogen receptor beta. Endocrinology.

[22] Valladares F, Frias I, Baez D, Garcia C, Lopez FJ, Fraser JD, Rodriguez Y, Reyes R, Diaz-Flores L, Bello AR (2006). Characterization of estrogen receptors alpha and beta in uterine leiomyoma cells. Fertil Steril.

[23] Collins F, MacPherson S, Brown P, Bombail V, Williams AR, Anderson RA, Jabbour HN, Saunders PT (2009). Expression of oestrogen receptors, ERalpha, ERbeta, and ERbeta variants, in endometrial cancers and evidence that prostaglandin F may play a role in regulating expression of ERalpha. BMC Cancer.

[24] Tsuboi Kouki, Nagatomo Takamasa, Gohno Tatsuyuki, Higuchi Toru, Sasaki Shunta, Fujiki Natsu, Kurosumi Masafumi, Takei Hiroyuki, Yamaguchi Yuri, Niwa Toshifumi, Hayashi Shin-ichi (2017). Single CpG site methylation controls estrogen receptor gene transcription and correlates with hormone therapy resistance. The Journal of Steroid Biochemistry and Molecular Biology.

[25] Yoshida T, Eguchi H, Nakachi K, Tanimoto K, Higashi Y, Suemasu K, Iino Y, Morishita Y, Hayashi S (2000). Distinct mechanisms of loss of estrogen receptor alpha gene expression in human breast cancer: methylation of the gene and alteration of trans-acting factors. Carcinogenesis.

[26] Tekmal RR, Liu YG, Nair HB, Jones J, Perla RP, Lubahn DB, Korach KS, Kirma N (2005). Estrogen receptor alpha is required for mammary development and the induction of mammary hyperplasia and epigenetic alterations in the aromatase transgenic mice. J Steroid Biochem Mol Biol.

[27] Watanabe J, Sasajima N, Aramaki A, Sonoyama K (2008). Consumption of fructo-oligosaccharide reduces 2,4-dinitrofluorobenzene-induced contact hypersensitivity in mice. Brit J Nutr.

[28] Russo J, Ao X, Grill C, Russo IH (1999). Pattern of distribution of cells positive for estrogen receptor alpha and progesterone receptor in relation to proliferating cells in the mammary gland. Breast Cancer Res Treat.

[29] Ewan KB, Oketch-Rabah HA, Ravani SA, Shyamala G, Moses HL, Barcellos-Hoff MH (2005). Proliferation of estrogen receptor-alpha-positive mammary epithelial cells is restrained by transforming growth factor-beta1 in adult mice. Am J Pathol.

[30] Band AM, Laiho M (2011). Crosstalk of TGF-beta and estrogen receptor signaling in breast cancer. J Mammary Gland Biol Neoplasia.

[31] Shepherd JA, Malkov S, Fan B, Laidevant A, Novotny R, Maskarinec G (2008). Breast density assessment in adolescent girls using dual-energy X-ray absorptiometry: a feasibility study. Cancer Epidemiol Biomark Prev.

[32] Javed A, Lteif A (2013). Development of the human breast. Semin Plast Surg.

[33] Yaghjyan L, Colditz GA, Collins LC, Schnitt SJ, Rosner B, Vachon C, Tamimi RM (2011). Mammographic breast density and subsequent risk of breast cancer in postmenopausal women according to tumor characteristics. J Natl Cancer Inst.

[34] Shepherd JA, Kerlikowske K, Ma L, Duewer F, Fan B, Wang J, Malkov S, Vittinghoff E, Cummings SR (2011). Volume of mammographic density and risk of breast cancer. Cancer Epidemiol Biomark Prev.

[35] Harris HR, Tamimi RM, Willett WC, Hankinson SE, Michels KB (2011). Body size across the life course, mammographic density, and risk of breast cancer. Am J Epidemiol.

[36] Gaskins AJ, Pereira A, Quintiliano D, Shepherd JA, Uauy R, Corvalan C, Michels KB (2017). Dairy intake in relation to breast and pubertal development in Chilean girls. Am J Clin Nutr.

[37] Boyd NF, Martin LJ, Bronskill M, Yaffe MJ, Duric N, Minkin S (2010). Breast tissue composition and susceptibility to breast cancer. J Natl Cancer Inst.

[38] Swerdlow AJ, De Stavola BL, Floderus B, Holm NV, Kaprio J, Verkasalo PK, Mack T (2002). Risk factors for breast cancer at young ages in twins: an international population-based study. J Natl Cancer Inst.

[39] MacMahon B, Cole P, Lin TM, Lowe CR, Mirra AP, Ravnihar B, Salber EJ, Valaoras VG, Yuasa S (1970). Age at first birth and breast cancer risk. Bull World Health Organ.

[40] Collaborative Group on Hormonal Factors in Breast C (2012). Menarche, menopause, and breast cancer risk: individual participant meta-analysis, including 118 964 women with breast cancer from 117 epidemiological studies. Lancet Oncol.

[41] Mayer C, Acosta-Martinez M, Dubois SL, Wolfe A, Radovick S, Boehm U, Levine JE (2010). Timing and completion of puberty in female mice depend on estrogen receptor alpha-signaling in kisspeptin neurons. Proc Natl Acad Sci U S A.

[42] Sano K, Nakata M, Musatov S, Morishita M, Sakamoto T, Tsukahara S, Ogawa S (2016). Pubertal activation of estrogen receptor alpha in the medial amygdala is essential for the full expression of male social behavior in mice. Proc Natl Acad Sci U S A.

[43] Peper JS, Brouwer RM, van Leeuwen M, Schnack HG, Boomsma DI, Kahn RS, Hulshoff Pol HE (2010). HPG-axis hormones during puberty: a study on the association with hypothalamic and pituitary volumes. Psychoneuroendocrinology.

[44] Cho YH, Yazici H, Wu HC, Terry MB, Gonzalez K, Qu MX, Dalay N, Santella RM (2010). Aberrant promoter hypermethylation and genomic hypomethylation in tumor, adjacent normal tissues and blood from breast cancer patients. Anticancer Res.

[45] Schwarzenbach H, Pantel K (2015). Circulating DNA as biomarker in breast cancer. Breast Cancer Res.

[46] Terry MB, McDonald JA, Wu HC, Eng S, Santella RM (2016). Epigenetic biomarkers of breast cancer risk: across the breast cancer prevention continuum. Adv Exp Med Biol.

[47] Galanter JM, Gignoux CR, Oh SS, Torgerson D, Pino-Yanes M, Thakur N, Eng C, Hu D, Huntsman S, Farber HJ, et al. Differential methylation between ethnic sub-groups reflects the effect of genetic ancestry and environmental exposures. elife. 2017;6. 10.7554/eLife.20532. 10.7554/eLife.20532 PMC5207770 28044981

[48] Biro FM, Greenspan LC, Galvez MP, Pinney SM, Teitelbaum S, Windham GC, Deardorff J, Herrick RL, Succop PA, Hiatt RA (2013). Onset of breast development in a longitudinal cohort. Pediatrics.

[49] Gonzalez L, Corvalan C, Pereira A, Kain J, Garmendia ML, Uauy R (2014). Early adiposity rebound is associated with metabolic risk in 7-year-old children. Int J Obes.

[50] Tanner J (1962). Growth at adolescence, 2nd ed.

[51] Pereira A, Garmendia ML, Gonzalez D, Kain J, Mericq V, Uauy R, Corvalan C (2014). Breast bud detection: a validation study in the Chilean growth obesity cohort study. BMC Womens Health.

[52] Shepherd JA, Herve L, Landau J, Fan B, Kerlikowske K, Cummings SR (2006). Clinical comparison of a novel breast DXA technique to mammographic density. Med Phys.

[53] Maskarinec G, Morimoto Y, Daida Y, Laidevant A, Malkov S, Shepherd JA, Novotny R (2011). Comparison of breast density measured by dual energy X-ray absorptiometry with mammographic density among adult women in Hawaii. Cancer Epidemiol.

[54] Adiotomre E, Summers L, Allison A, Walters SJ, Digby M, Broadley P, Lang I, Morrison G, Bishop N, Arundel P (2017). Diagnostic accuracy of DXA compared to conventional spine radiographs for the detection of vertebral fractures in children. Eur Radiol.

[55] Ye X, Kuklenyik Z, Needham LL, Calafat AM (2005). Automated on-line column-switching HPLC-MS/MS method with peak focusing for the determination of nine environmental phenols in urine. Anal Chem.

[56] Silva MJ, Samandar E, Preau JL, Reidy JA, Needham LL, Calafat AM (2007). Quantification of 22 phthalate metabolites in human urine. J Chromatogr B Analyt Technol Biomed Life Sci.

[57] Boeniger MF, Lowry LK, Rosenberg J (1993). Interpretation of urine results used to assess chemical-exposure with emphasis on creatinine adjustments - a review. Am Ind Hyg Assoc J.

[58] Teass AW, Biagini RE, DeBord G, Hull RD. Application of Biological Monitoring Methods. NIOSH Man Anal Method. Cincinnati: National Institute for Occupational Safety and Health Division of Physical Sciences and Engineering; 1998.

[59] Cediel G, Corvalan C, Aguirre C, de Romana DL, Uauy R (2016). Serum 25-Hydroxyvitamin D associated with indicators of body fat and insulin resistance in prepubertal Chilean children. Int J Obes.

