# Supplementary material for: Inverse association between estrogen receptor-α DNA methylation and breast composition in adolescent Chilean girls
Source: Clin Epigenetics. 2018 Oct 4;10:122. doi: 10.1186/s13148-018-0553-5 (PMC6172836; doi:10.1186/s13148-018-0553-5)
Supplement: Supplementary file 1 — Figure S1. Correlation across CpG sites at B2 and B4 (Spearman rho = 0.65–98). Table S1: Primer sequences and positions. (DOCX 592 kb) [file 13148_2018_553_MOESM1_ESM.docx]

**Supplement:**

**Table S1: Primer sequences and positions**

| Gene | Primers | CpG coverage | CpG positions |
| --- | --- | --- | --- |
| ERα | F: 5'-GGGTAGGGTTGGGGTTAGAG-3'  R: 5'-Biotin- ATCCCCRCAAAACAAAAAACTCAAA-3'  S: 5'-GGGTTGGGGTTAGAGT-3' | 10 | Chr6: 151,807,783  Chr6: 151,807,785  Chr6: 151,807,790  Chr6: 151,807,793  Chr6: 151,807,802  Chr6: 151,807,807  Chr6: 151,807,810  Chr6: 151,807,822  Chr6: 151,807,849  Chr6: 151,807,852 |

**Figure S1:** **Correlation across CpG sites at B2 and B4** (Spearman rho= 0.65-98).
